# Outcomes After Respiratory Virus Detection Prior to Pediatric Solid Organ Transplantation

**DOI:** 10.1111/petr.70532

**Published:** 2026-11-06

**Authors:** Mallory Go, Mario M. Landa, Joanna Malec, Molly Schnieders, William J. Muller

**Affiliations:** ^1^ Department of Epidemiology Brown University School of Public Health Providence Rhode Island USA; ^2^ Division of Infectious Diseases Ann & Robert H. Lurie Children's Hospital of Chicago Chicago Illinois USA; ^3^ Stanley Manne Children's Research Institute Chicago Illinois USA; ^4^ Department of Pediatrics Northwestern University Feinberg School of Medicine Chicago Illinois USA

**Keywords:** pediatric solid organ transplant, pulmonary outcomes, respiratory virus infection

## Abstract

**Background:**

Respiratory viral infections are associated with increased pulmonary morbidity in immunocompromised populations. It is not known whether pediatric solid organ transplant (SOT) recipients who test positive for respiratory viruses prior to transplant have increased risk of adverse pulmonary outcomes.

**Methods:**

We conducted a single‐center retrospective study of pediatric SOT recipients tested for respiratory viruses by multiplex PCR in the 30 days prior to their transplant.

**Results:**

Fourteen of 45 recipients who were tested had a virus identified, one of whom tested negative on a subsequent repeat test prior to transplant. The remaining 13 were matched 1:2 to control transplant recipients matched by age at transplant, organ transplanted, and date of transplant. No differences in adverse pulmonary outcomes were identified up to 90 days post‐SOT. A similar comparison of patients testing positive to 31 patients with negative respiratory virus testing also did not identify any differences in adverse pulmonary outcomes.

**Conclusions:**

Among pediatric SOT recipients who underwent clinically indicated respiratory viral testing within 30 days prior to transplant, we did not observe a clear excess of post‐transplant pulmonary morbidity. Larger multicenter studies are needed to confirm this observation.

## 1 Introduction

Respiratory virus infections (RVIs) are the most frequent type of infection in the pediatric population and contribute significantly to the burden of disease in infants and young children globally [1, 2, 3]. In the general pediatric population, RVIs are generally self‐limiting, though they may rarely lead to severe disease or even mortality [1, 2, 3]. Immunocompromised patients including recipients of solid organ transplants (SOT) or stem cell transplants (SCT) are at higher risk of morbidity and mortality [4, 5]. Patients undergoing SCT are commonly screened for RVI prior to undergoing conditioning, with transplant often delayed in those with viruses detected due to worse outcomes [6].

Although data on complications associated with diagnosis of RVI after pediatric SOT are comparable to those after SCT [4, 5], it is not known whether detection of RVI prior to SOT is associated with worse outcomes after transplant. Most recipients of SOT receive organs from deceased donors, which affects the risk–benefit determination for deferring transplant relative to SCT, as the next opportunity to receive an organ is not predictable. SCT recipients may have more compromised immunity prior to transplant than SOT candidates, given the different indications for the two interventions. Additionally, immunosuppressive regimens for SCT generally suppress immunity more broadly than those used in SOT, which are lymphocyte‐directed. Accordingly, it is not clear whether RVI prior to SOT is as concerning as it has been shown to be in those undergoing SCT.

Lurie Children's is a major pediatric solid organ transplant center, having completed more than 300 transplants since 2021. We conducted a single center retrospective study of our pediatric SOT recipients tested for RVI within 30 days of SOT to investigate the epidemiology and outcomes in those testing positive.

## 2 Methods

### 2.1 Patient Population

This single center study analyzed data from SOT recipients transplanted between January 1, 2021, and October 31, 2025 at Ann & Robert H. Lurie Children's Hospital of Chicago, who were under age 18 years at the time of transplant. Case patients were transplanted prior to December 31, 2024 and were tested for RVI in the 30 days prior to transplant. Follow‐up for respiratory outcomes was for 90 days after transplant, with death or graft loss followed for up to 1 year after transplant. Control patients used for some comparisons were matched with case patients by age at transplant, organ transplanted, and date of transplant; to ensure adequate matching, some control transplants occurred through October 31, 2025. Data were collected retrospectively from electronic medical records. Patients who received a heart, liver, kidney, small bowel, or multivisceral transplant at Lurie Children's were eligible to be included, as were patients undergoing a second transplant. Patients followed at Lurie Children's who received their transplants at other centers were excluded.

Records from SOT recipients satisfying the above criteria were searched to identify those patients who were tested for RVI within a 30‐day window prior to their transplant. We included only tests involving multiplex nucleic acid testing of respiratory secretions with a panel that identified 19 targets of respiratory infections (Table 1). We excluded testing that involved more limited detection of respiratory viruses, as those tests were commonly used for screening and most high‐risk patients at our institution (including transplant recipients or candidates) with concern for infection will be tested with the complete panel. We excluded patients from case–control analyses if they had a positive test within the window but a subsequent negative test prior to transplant.

**TABLE 1:** Targets detected on RVI panel used in this study.

Targets
Adenovirus
*Bordetella pertussis*
*Bordetella parapertussis*
*Chlamydophila pneumoniae*
Coronavirus 229E
Coronavirus HKU1
Coronavirus NL63
Coronavirus OC43
SARS CoV‐2
Human Metapneumovirus
Human Rhinovirus/Enterovirus
Influenza A
Influenza B
*Mycoplasma pneumoniae*
Parainfluenza Virus 1
Parainfluenza Virus 2
Parainfluenza Virus 3
Parainfluenza Virus 4
Respiratory Syncytial Virus (RSV)

Individual records were reviewed to confirm that testing was done for symptomatic reasons (i.e., not for screening); only tests sent for concern of possible infection were included as cases. We categorized those with respiratory symptoms as having upper respiratory tract infection (URTI) or lower respiratory tract infection (LRTI) based on criteria used in prior publications. The case definition for LRTI included at least one documented physical examination finding localizing to the lower respiratory tract, and at least one objective clinical sign of severe respiratory disease [7, 8]. Physical findings included documentation of rhonchi, rales, crackles, or wheeze. Objective clinical signs included at least one documented respiratory rate above the upper limit of normal for age, at least one documented measurement of oxygen saturation less than 95%, or at least one of the following: retractions, grunting, nasal flaring, acute hypoxic or ventilatory failure, intercostal, subcostal, or supraventricular retractions, or (in children under 2 years of age) new‐onset apnea or dehydration due to respiratory distress requiring IV hydration. The case definition for URTI included failure to meet criteria for LRTI, and documentation of at least one sign of an upper respiratory illness reported by a caregiver or health care provider, including cough, congestion, rhinorrhea, and/or increased work of breathing within 72 h of the respiratory virus test [4]. Most tested patients had cough, rhinorrhea, or other respiratory symptoms, but some presented with fever without a clear source or new‐onset seizure, and were tested due to clinical concern for infection. For some analyses, comparisons were made between patients who tested positive for a respiratory virus and those who tested negative.

### 2.2 Case–Control Analyses

Two comparisons were made for this study. The primary analysis included case patients as defined above with a positive RVI test, compared with matched controls who were not tested for a respiratory virus. The intent of this comparison was to determine whether any adverse pulmonary outcomes were more common in those testing positive for RVI than in control patients undergoing transplant without concern for RVI, with the goal of analyzing potential risk factors associated with outcomes of interest after a positive test for RVI. Controls were matched 2:1 to case patients based on age at transplant (within 14 months), organ transplanted, and date of transplant (within 20 months), but were not tested for RVI in the 30 days prior to transplant.

An additional similar comparison was made between case patients who tested positive for a respiratory virus within 30 days of SOT and patients who had a multiplex test performed within 30 days of transplant that resulted negative for a respiratory virus. The intent of this comparison was to determine whether any adverse pulmonary outcomes were more common in those testing positive for RVI than in patients undergoing transplant for whom there was concern for RVI, but an infectious cause was not identified. Since this comparison involved testing conducted for clinical concern, matching was not able to be done.

### 2.3 Covariates and Outcome Measures

Individual charts for case and control patients were reviewed to determine their respiratory status and symptoms in the 30‐day period prior to transplant. Need for ventilatory support prior to transplant due to underlying illness was recorded, ranging from supplemental oxygen to mechanical ventilation. Post‐transplant outcome measures related to respiratory status included days to extubation after transplant (or days to transition to a home ventilator, for patients requiring home ventilatory support at discharge) and days to return to room air and/or stable home ventilator settings. Additional complications chosen based on prior studies of respiratory viral infections in stem cell [6] and solid organ transplant [5] recipients included tracheostomy at 90 days after transplant, need for bronchoscopy within 90 days after transplant, and need for ventilatory support at 90 days post‐transplant. Additional clinical outcomes relevant to the potential contribution of a pre‐transplant RVI to a more complicated post‐transplant course included days from transplant to ICU discharge and days from transplant to hospital discharge. We also assessed graft loss or mortality within 1 year of transplant.

Potential factors which could contribute to worse outcomes from infection were also collected from the electronic medical record, including induction immunosuppressive regimen, CMV and EBV serostatus, and donor status (living or deceased donor).

### 2.4 Statistical Analysis

Given the small sample size and low frequency of study outcomes, analyses were prespecified as primarily descriptive and exploratory. Categorical variables were summarized as counts and percentages. Two prespecified pairwise comparisons were performed: patients testing positive versus matched controls who were not tested, and patients testing positive versus patients testing negative.

Binary categorical variables were compared between groups using Fisher's exact test because of sparse cell counts. Odds ratios (ORs) and exact 95% confidence intervals (CIs) were estimated from two‐by‐two contingency tables using conditional maximum‐likelihood estimation. Estimates were interpreted cautiously given the limited sample size and imprecision of several outcomes. For categorical variables with more than two levels, global *p*‐values were estimated using Fisher's exact test with Monte Carlo simulation based on 10 000 replicates. For descriptive purposes, category‐specific ORs and exact 95% CIs were calculated using separate two‐by‐two contingency tables comparing each category with the designated reference category.

Continuous variables were summarized as median and interquartile range (IQR) and compared using two‐sided Wilcoxon rank‐sum tests for both prespecified pairwise comparisons. No multivariable regression modeling was performed because the number of exposed patients and outcome events was insufficient for stable adjusted analyses.

Statistical analyses were performed using RStudio [9].

### 2.5 Guidelines and Ethics

This study was approved by the Institutional Review Board of Ann & Robert H. Lurie Children's Hospital of Chicago (STUDY00000050).

## 3 Results

### 3.1 Population

There were 282 transplants in 278 individual patients at our center between January 1, 2021, and December 31, 2024. We identified 45 patients who underwent RVI testing within 30 days prior to transplant who were tested for diagnostic purposes. More than 3/4 of tests were in patients under age 6, in which symptomatic respiratory illness is generally more common [10]. Most tests were in recipients of heart or liver transplants, who tend to be younger in age.

### 3.2 Case–Control Analysis

Of the 45 patients undergoing RVI testing in the 30‐day period prior to transplant, 14 had a virus identified (31% of those tested and 5% of the total number of transplants). All were either heart or liver recipients, and only one was older than 12 years at the time of transplant (Table 2). All patients who tested positive met criteria for URTI (none met criteria for LRTI). One patient who tested positive twice for rhinovirus/enterovirus 19 and 11 days prior to SOT had a subsequent negative test 4 days prior to SOT and was excluded from case–control analyses. The remaining RVI‐positive case patients were compared with control patients matched for age, organ transplanted, and date of transplant who did not undergo RVI testing (Table 2). There were no statistically significant differences in demographic or transplant‐related characteristics of cases compared to controls, including sex, race, ethnicity, induction immunosuppression, CMV or EBV serostatus, and whether the donor was deceased or living. No control patients met criteria for URTI or LRTI. Accordingly, patients who tested positive for RVI were more likely to have respiratory symptoms during the 30‐day period prior to transplant compared to matched control patients (Table 2). There was no between‐group difference in need for respiratory support (oxygen and/or ventilator dependence) prior to transplant.

**TABLE 2:** Characteristics of patients testing positive for RVI within 30 days prior to SOT, compared with their matched controls.

Patients	Patients testing positive	Controls	
Total	13	26	
Sex			*p*
Female	9	13	0.318
Male	4	13	
Race			
Asian	0	0	0.330
Black or African American	5	8	
Multiple or Other	4	4	
White	4	14	
Ethnicity			
Hispanic or latine	3	4	0.666
Not hispanic or latine	10	22	
Declined or unknown	0	0	
**Transplant characteristics**			
Organ transplanted			
Heart	9	18	1.000
Liver	4	8	
Kidney	0	0	
Multivisceral	0	0	
Year of transplant			
2021	2	6	0.674
2022	3	2	
2023	3	7	
2024	5	9	
2025	0	2	
Age at transplant (yrs)			
0–2	7	16	0.872
2–6	5	8	
6–12	0	0	
12–20	1	2	
Induction immunosuppression			
Basiliximab	0	0	1.000
ATG	8	16	
Other	5	10	
CMV serostatus			
CMV R+	5	11	0.927
CMV R−/D+	1	3	
CMV R−/D−	7	11	
CMV status unknown	0	1	
EBV serostatus			
EBV R+	5	9	0.943
EBV R−/D+	3	8	
EBV R−/D−	5	8	
EBV status unknown	0	1	
Deceased Donor			
Yes	11	23	1.000
No	2	3	
**RVI characteristics**			**OR (95% CI)**
Respiratory symptoms reported			
Yes	11	3	35.61 (4.88–486.33)
No	2	23	*p* < 0.001
Oxygen and/or ventilator dependence pre‐RVI testing/within 30 days SOT			
Yes	4	7	1.00 (0.17–5.11)
No	9	19	*p* = 1.000
**Outcomes**			
Days from SOT to extubation or transition to a home ventilator			*p*
Median [IQR]	2 [1–17]	3 [1–7.8]	0.609
Days from SOT to return to room air and/or stable home ventilator settings			
Median [IQR]	15 [4–22]	9.5 [4–13]	0.622
Days from SOT to discharge from ICU			
Median [IQR]	25 [16–30]	18 [12.5–32.2]	0.561
Days from SOT to discharge from hospital			
Median [IQR]	27 [17–30]	27.5 [17.2–34.5]	1.000
Oxygen and/or ventilator dependence 90 days post‐SOT			OR (95% CI)
Yes	2	2	2.13 (0.14–33.08)
No	11	24	*p* = 0.589
Bronchoscopy within 90 days of SOT			
Yes	1	2	1.00 (0.02–21.07)
No	12	24	*p* = 1.000
Tracheostomy within 90 days of SOT^a^			
Yes	1	1	2.04 (0.02–169.71)
No	12	25	*p* = 1.000
Graft loss within 1 year of SOT^b^			*p*
Yes	0	0	1.000
No	13	23	
Mortality within 1 year of SOT			
Yes	0	3	0.538
No	13	23	

^a^ In both cases, the patient had a tracheostomy prior to transplant.

^b^ Patients who died within 1 year of transplant were excluded.

Post‐surgery pulmonary outcomes did not differ statistically between positive cases and controls, including no statistically significant differences between groups in days from transplant to extubation, days from transplant to return to room air or stable home ventilator settings, need for oxygen/mechanical ventilation, need for bronchoscopy within the 3 months after transplantation, or need for tracheostomy within the 3 months after transplantation. Days from transplant to discharge from ICU or from the hospital also did not differ between groups. There were no statistical differences in one‐year post‐transplant survival, and no patients had graft loss in the year after transplant in either group.

Since all positive tests involved either heart or liver transplant recipients, an exploratory analysis for continuous outcomes was done by organ type (Supplementary Table 1). Small numbers precluded analyses for binary outcome measures (need for oxygen/mechanical ventilation, need for bronchoscopy within the 3 months after transplantation, or need for tracheostomy within the 3 months after transplantation). There were no statistical differences between groups based on organ transplanted in days from transplant to extubation, days from transplant to return to room air or stable home ventilator settings, or days from transplant to discharge from either the ICU or the hospital.

### 3.3 Comparison of Patients Testing Positive for RVI to Those Testing Negative

We also compared patients testing positive for RVI within 30 days prior to transplant to those who tested negative (Table 3). Patients who tested positive for RVI were more likely to have respiratory symptoms during the 30‐day period prior to transplant, compared to patients who tested negative. Twelve of the 31 patients who tested negative met criteria for URTI, with others tested due to concern for infection without obvious respiratory symptoms (usually fever). None met criteria for LRTI. There was no between‐group difference in need for respiratory support (oxygen and/or ventilator dependence prior to transplant).

**TABLE 3:** Characteristics of patients tested for RVI within 30 days prior to SOT, comparing those with a positive test to those with negative testing.^a^

Patients	Patients testing positive	Patients testing negative	
Total	13	31	
Sex			*p*
Female	9	14	0.194
Male	4	17	
Race			
Asian	0	1	0.940
Black or African American	5	9	
Multiple or Other	4	12	
White	4	9	
Ethnicity			
Hispanic or latine	3	6	1.000
Not Hispanic or latine	10	24	
Declined or unknown	0	1	
**Transplant characteristics**			
Organ Transplanted			
Heart	9	14	0.577
Liver	4	13	
Kidney	0	3	
Multivisceral	0	1	
Year of Transplant			
2021	2	7	0.859
2022	3	4	
2023	3	9	
2024	5	11	
2025	0	0	
Age at Transplant (yrs)			
0–2	7	13	0.609
2–6	5	9	
6–12	0	5	
12–20	1	4	
Induction immunosuppression			
Basiliximab	0	5	0.449
ATG	8	16	
Other	5	10	
CMV serostatus			
CMV R+	5	4	0.045
CMV R−/D+	1	12	
CMV R−/D−	7	15	
CMV status unknown	0	0	
EBV serostatus			
EBV R+	5	9	0.475
EBV R−/D+	3	13	
EBV R−/D−	5	7	
EBV status unknown	0	2	
Deceased donor			
Yes	11	27	1.000
No	2	4	
**RVI characteristics**			OR (95% CI)
Respiratory symptoms reported			
Yes	11	16	4.98 (0.87–53.58)
No	2	15	*p* = 0.050
Oxygen and/or ventilator dependence pre‐RVI testing/within 30 days SOT			
Yes	4	15	0.48 (0.09–2.20)
No	9	16	*p* = 0.335
**Outcomes** ^b^			
Days from SOT to extubation or transition to a home ventilator			*p*
Median [IQR]	2 [1–17]	3 [1–17]	0.948
Days from SOT to return to room air and/or stable home ventilator settings			
Median [IQR]	15 [4–22]	20 [5.5–49]	0.403
Days from SOT to discharge from ICU			
Median [IQR]	25 [16–30]	25 [14–56]	0.767
Days from SOT to discharge from hospital			
Median [IQR]	27 [17–30]	41 [24.5–63.5]	0.207
Oxygen and/or ventilator dependence 90 days post‐SOT			OR (95% CI)
Yes	2	11	0.32 (0.03–1.91)
No	11	19	*p* = 0.280
Bronchoscopy within 90 days of SOT			
Yes	1	10	0.17 (0.00–1.50)
No	12	20	*p* = 0.129
Tracheostomy within 90 days of SOT			
Yes	1	0	not estimable
No	12	30	*p* = 0.302
Graft loss within 1 year of SOT^c^			*p*
Yes	0	0	1.000
No	13	29	
Mortality within 1 year of SOT			
Yes	0	2	1.000
No	13	29	

^a^ One patient was tested three times for RVI prior to transplant, with the first two tests positive for rhinovirus/enterovirus 19 and 11 days prior to SOT and the third test negative 4 days prior to SOT; this patient was excluded from this Table.

^b^ One patient who tested negative for RVI died within 90 days of transplant and is not included in the 90‐day respiratory outcomes.

^c^ Patients who died within 1 year of transplant were excluded.

Pulmonary outcomes did not differ between patients testing positive and those testing negative, including no statistically significant differences between groups in days from transplant to extubation, days from transplant to return to room air or stable home ventilator settings, need for oxygen/mechanical ventilation, need for bronchoscopy within the 3 months after transplantation, or need for tracheostomy within the 3 months after transplantation. Days from transplant to discharge from ICU or from the hospital also did not differ between groups. There were no statistical differences in one‐year post‐transplant survival, and no patients had graft loss in the year after transplant in either group. Similarly, there were no statistical differences based on organ transplanted (Supplementary Table 2), again noting the caveat that overall numbers were small.

### 3.4 Microbiology

At least one virus was detected in 14 patients tested for RVI in the 30 days prior to SOT (Table 4). Rhinovirus/enterovirus was the most common virus detected. Four patients had two viruses detected; three co‐detections involved rhinovirus/enterovirus, once with adenovirus and twice with SARS‐CoV‐2, and one co‐detection involved adenovirus with SARS‐CoV‐2. One patient tested three times for RVI prior to transplant had two tests positive for rhinovirus/enterovirus 19 and 11 days prior to SOT, with a negative test 4 days prior to SOT. Recipients of heart transplants were more likely to have a virus detected, as were younger patients (Supplementary Table 3). Nine of the 14 patients who tested positive prior to transplant had at least one positive test for the same or a different respiratory virus in the 90 days after their transplant (Table 4), with no clear association with negative outcomes.

**TABLE 4:** Viral testing post‐transplant in patients who tested positive in the 30 days prior to SOT.^a^

Episode ID	Virus detected prior to SOT	Days prior to SOT	Viruses detected in the 90 days after SOT	Days after SOT	Negative respiratory outcome at 90 days?^b^
18	Rhinovirus/enterovirus	16	Rhinovirus/enterovirus Rhinovirus/enterovirus Adenovirus	9 44 86	Yes
20	Rhinovirus/enterovirus	27	Not tested		No
21	Adenovirus Rhinovirus/enterovirus	1	Rhinovirus/enterovirus	25	No
24	Respiratory syncytial virus	11	Negative	14	No
37	Influenza A	24	Negative Negative	11 63	No
41	SARS‐CoV‐2 Rhinovirus/enterovirus	20	Rhinovirus/enterovirus	4	No
44	Adenovirus	5	Negative Rhinovirus/enterovirus RSV, rhinovirus/enterovirus	19 22 26	No
47	Respiratory syncytial virus	20	Rhinovirus/enterovirus Rhinovirus/enterovirus Rhinovirus/enterovirus	28 55 86	No
52	Rhinovirus/enterovirus	19	Rhinovirus/enterovirus Rhinovirus/enterovirus	7 9	No
53	Adenovirus SARS‐CoV‐2	9	SARS‐CoV‐2 Negative SARS‐CoV‐2	4 11 21	Yes
56	Rhinovirus/enterovirus	11	SARS‐CoV‐2	6	No
59	Rhinovirus/enterovirus	20	Negative Negative Negative	20 33 37	Yes
60	Rhinovirus/enterovirus	7	Rhinovirus/enterovirus Negative Negative	12 23 37	No

^a^ One patient was tested three times for RVI prior to transplant, with the first two tests positive for rhinovirus/enterovirus 19 and 11 days prior to SOT and the third test negative 4 days prior to SOT; this patient was excluded from this Table and had 1 additional negative Test 11 days after SOT.

^b^ Negative respiratory outcomes included oxygen and/or ventilator dependence 90 days post‐SOT, bronchoscopy within 90 days of SOT, or tracheostomy within 90 days of SOT.

## 4 Discussion

Respiratory viral infections in immunocompromised children including recipients of solid organ transplants are associated with adverse outcomes, particularly when infection occurs in the weeks after transplant [5]. Although detection of respiratory viruses prior to allogeneic stem cell transplantation has been found to confer a risk for adverse pulmonary outcomes [6], similar data are lacking for patients undergoing solid organ transplantation. We found that positive testing for RVI prior to SOT was not associated with adverse pulmonary outcomes, including need for oxygen/mechanical ventilation or need for bronchoscopy within the 3 months after transplantation.

Patients included in this study underwent testing for RVI when the treating clinical team had concerns for infection, though only about a third of tests detected a virus. Most patients were tested due to the presence of symptoms of respiratory infection such as cough or rhinorrhea, though none met criteria for LRTI. Some patients were tested as part of a broad evaluation for infection after presenting with symptoms such as unexplained fever or new onset seizure, though none of these had a positive test. As expected, patients testing positive for respiratory viruses prior to SOT were significantly more likely to have respiratory symptoms documented when compared to matched untested controls matched on age at transplant, organ transplanted, and date of transplantation (Table 2). However, no difference in outcomes was observed. Similarly, patients who tested positive for respiratory viruses prior to SOT were slightly (though not statistically significantly) more likely to have respiratory symptoms documented prior to testing when compared to patients who were tested but did not have a respiratory virus identified (Table 3), but pulmonary outcomes also did not differ between these groups. Finally, in no cases of a positive test did we note that a patient progressed to LRTI between the positive test and after their SOT; progression from URTI to LRTI is a concerning outcome after pre‐transplant detection of respiratory viruses in the SCT setting [6]. Together, these observations suggest that detection of a respiratory virus prior to solid transplantation may not be as concerning as it is prior to stem cell transplantation [6, 11].

There are clear limitations to this study. As a single center study, there may be center‐specific differences in management of SOT candidates and recipients that influence outcomes, complicating any extrapolation to other settings. Although our center has a large volume of pediatric solid organ transplants, the overall number of patients analyzed was small, resulting in some imprecision in the analysis. However, none of the examined outcomes met the conventional threshold for statistical significance. Second, due to the relatively small patient population analyzed, our analysis grouped the detection of any respiratory virus in the positive group, and we are accordingly unable to determine whether any individual virus may be of greater concern for post‐SOT complications. Most of our positive tests prior to SOT involved rhinovirus/enterovirus, which, although often considered to be less severe than other respiratory viral pathogens, has independently been associated with worse outcomes—including mortality—in the stem cell transplant population [6]. Indeed, two of the three patients with pulmonary morbidity at 90 days post‐SOT tested positive for rhinovirus/enterovirus prior to SOT (Table 4). Lastly, all of our patients with positive RVI tests received either heart or liver transplants, limiting our ability to generalize our observations to other transplant types.

Our small study of positive tests for RVI prior to pediatric SOT serves to support the hypothesis that for at least some patients, the risk–benefit balance for transplant within 30 days of a positive test for a respiratory virus may favor proceeding. A prior survey of pediatric infectious diseases experts in the care of solid organ transplant patients supported a recommendation against delaying an urgent transplant in the setting of URTI, though a majority recommended brief delay if a virus was identified [12]. Our small retrospective study would not provide sufficient basis to affect individualized decision making for transplant candidates with URTI, but does provide preliminary reassurance that for some symptomatic patients, proceeding with transplant following a prior diagnosis with a respiratory virus did not increase the risk for adverse outcomes. Additionally, our review of the records did not identify any transplants that were delayed due to a positive RVI test. Preliminary data from an ongoing multicenter study reported that it is not uncommon to detect rhinovirus/enterovirus from pediatric SOT recipients at the time of transplant [13], and this ongoing study is anticipated to provide additional information on outcomes of patients with these infections prior to transplant.

## 5 Conclusions

Respiratory viruses were detected in symptomatic pediatric patients within 30 days prior to solid organ transplant, with no clear associated adverse outcomes. Larger multicenter studies are needed to confirm the observation that pre‐SOT detection of a respiratory virus in this patient population does not confer an unacceptable risk for adverse outcomes after SOT.

## Author Contributions

M.G. and W.J.M. began the project and devised the research idea and project plan. M.G., M.M.L., M.S., and J.M. reviewed charts and performed data entry and cleaning. M.G. and M.M.L. performed data analysis with W.J.M. providing critical feedback, input, and consultation. All authors contributed to the writing of the manuscript. All authors read and approved the final manuscript for publication.

## Funding

This work was funded through the support of the Washington Family and the Ted Emerson Miller Research Scholar Fund.

## Disclosure

W.J.M. has served as site PI for clinical trials for Ansun Biopharma, Astellas Pharma, AstraZeneca, Eli Lilly and Company, Enanta Pharmaceuticals, F. Hoffmann‐La Roche Ltd. (Roche), Gilead Sciences, Melinta Therapeutics Inc., Merck Sharpe & Dohme, Moderna, Nabriva Therapeutics, plc, Paratek Pharmaceuticals Inc., Pfizer, and Tetraphase Pharmaceuticals Inc., and as a consultant or advisory board member for Astellas Pharma, AstraZeneca, Enanta Pharmaceuticals, Finley Law Firm, P.C., ProventionBio, and Sanofi Pasteur.

## Conflicts of Interest

The authors declare no conflicts of interest.

## Supporting information


**Supplementary Table 1.** Outcomes by organ type for patients testing positive for RVI within 30 days prior to SOT, compared with their matched controls.
**Supplementary Table 2**. Outcomes by organ type for patients testing positive for RVI within 30 days prior to SOT, compared with those testing negative for RVI.
**Supplementary Table 3**. Respiratory viruses detected in case patients.

## Data Availability

The data that support the findings of this study are available from the corresponding author upon reasonable request.
