# Supplementary material for: Outcomes After Respiratory Virus Detection Prior to Pediatric Solid Organ Transplantation
Source: Pediatr Transplant. 2026 Nov 6;30(11):e70532. doi: 10.1111/petr.70532 (PMC13743685; doi:10.1111/petr.70532)
Supplement: Supplementary file 1 [file PETR-30-e70532-s001.docx]

**Supplementary Table 1.**	Outcomes by organ type for patients testing positive for RVI within 30 days prior to SOT, compared with their matched controls

| **Heart transplant recipients** | **Patients testing positive (N=9)** | **Controls**  **(N=18)** |  |
| --- | --- | --- | --- |
| **Days from SOT to extubation or transition to a home ventilator** | | | **P** |
| Median [IQR] | 2.0 [1.0–7.0] | 1.0 [1.0–5.8] | 0.545 |
| **Days from SOT to return to room air and/or stable home ventilator settings** | | |  |
| Median [IQR] | 4.0 [4.0–16.0] | 7.0 [4.0–22.0] | 0.856 |
| **Days from SOT to discharge from ICU** | | |  |
| Median [IQR] | 27.0 [16.0–30.0] | 28.5 [17.5–49.5] | 0.625 |
| **Days from SOT to discharge from hospital^a^** | | |  |
| Median [IQR] | 27.0 [16.0–30.0] | 28.5 [17.5–72.8] | 0.662 |
| **Liver transplant recipients** | **Patients testing positive (N=4)** | **Controls**  **(N=8)** |  |
| **Days from SOT to extubation or transition to a home ventilator** | | | **P** |
| Median [IQR] | 15.0 [9.8–20.5] | 6.5 [4.5–8.2] | 0.203 |
| **Days from SOT to return to room air and/or stable home ventilator settings** | | |  |
| Median [IQR] | 20.0 [13.5–25.2] | 12.5 [9.0–13.0] | 0.199 |
| **Days from SOT to discharge from ICU** | | |  |
| Median [IQR] | 21.5 [16.2–28.2] | 12.0 [8.2–14.2] | 0.074 |
| **Days from SOT to discharge from hospital** | | |  |
| Median [IQR] | 28.5 [26.2–34.8] | 22.5 [17.8–29.2] | 0.234 |

^a^-Note: most heart transplant recipients in our hospital remain in cardiac intensive care for their entire hospital stay.

**Supplementary Table 2.**	Outcomes by organ type for patients testing positive for RVI within 30 days prior to SOT, compared with those testing negative for RVI

| **Heart transplant recipients** | **Patients testing positive (N=9)** | **Patients testing negative (N=14)** |  |
| --- | --- | --- | --- |
| **Days from SOT to extubation or transition to a home ventilator** | | | **P** |
| Median [IQR] | 2.0 [1.0–7.0] | 2.0 [1.0–3.0] | 0.871 |
| **Days from SOT to return to room air and/or stable home ventilator settings** | | |  |
| Median [IQR] | 4.0 [4.0–16.0] | 33.0 [5.2–55.8] | 0.376 |
| **Days from SOT to discharge from ICU** | | |  |
| Median [IQR] | 27.0 [16.0–30.0] | 41.5 [24.2–63.8] | 0.231 |
| **Days from SOT to discharge from hospital^a^** | | |  |
| Median [IQR] | 27.0 [16.0–30.0] | 45.5 [24.2–63.8] | 0.231 |
| **Liver transplant recipients** | **Patients testing positive (N=4)** | **Patients testing negative (N=13)** |  |
| **Days from SOT to extubation or transition to a home ventilator** | | | **P** |
| Median [IQR] | 15.0 [9.8–20.5] | 16.0 [4.0–23.0] | 0.821 |
| **Days from SOT to return to room air and/or stable home ventilator settings** | | |  |
| Median [IQR] | 20.0 [13.5–25.2] | 20.0 [7.0–25.0] | 0.821 |
| **Days from SOT to discharge from ICU** | | |  |
| Median [IQR] | 21.5 [16.2–28.2] | 20.0 [11.0–36.0] | 0.955 |
| **Days from SOT to discharge from hospital** | | |  |
| Median [IQR] | 28.5 [26.2–34.8] | 31.0 [25.0–55.0] | 0.610 |

^a^-Note: most heart transplant recipients in our hospital remain in cardiac intensive care for their entire hospital stay.

**Supplementary Table 3.**	Respiratory viruses detected in case patients

| **Virus detected** | **Number of detections** | **Organ received (N)** | **Age range, years (N)** | |
| --- | --- | --- | --- | --- |
| Rhinovirus/enterovirus | 8 | Heart: 5  Liver: 3 | 0-2:  2-6:  6-12:  12-20: | 4  4  0  0 |
| Adenovirus | 3 | Heart: 3 | 0-2:  2-6:  6-12:  12-20: | 2  1  0  0 |
| SARS-CoV-2 | 2 | Heart: 2 | 0-2:  2-6:  6-12:  12-20: | 1  1  0  0 |
| Respiratory syncytial virus | 2 | Heart: 2 | 0-2:  2-6:  6-12:  12-20: | 0  1  0  1 |
| Influenza A | 1 | Liver: 1 | 0-2:  2-6:  6-12:  12-20: | 1  0  0  0 |
